# T cells in ARAP-deficient mice present defective T cell receptor signaling and reduced severity in an experimentally-induced autoimmune disease

**DOI:** 10.3389/fimmu.2025.1556616

**Published:** 2025-04-08

**Authors:** Jee-Hae Kim, Seung Hee Jung, Chohee Park, Jong Ran Lee

**Affiliations:** Department of Life Science, College of Natural Sciences, Ewha Womans University, Seoul, Republic of Korea

**Keywords:** T cell receptor signaling, adaptor protein, ARAP, integrin-mediated adhesion, autoimmune disease

## Abstract

We previously reported a novel adaptor protein, ARAP, required for T cell receptor signaling and integrin-mediated adhesion. The present study investigates further the role of ARAP in T cell biology using mice with an *ARAP* gene deficiency. Similar to wild-type mice, ARAP-deficient mice participate in normal breeding and immune cell development. Similar defects were observed in the T cell receptor signaling and adhesion of ARAP-deficient mice, as shown in previous studies investigating ARAP-suppressed Jurkat T cells. ARAP deficiencies analyzed *in vivo* presented a less severe clinical course of experimental autoimmune encephalomyelitis (EAE) following immunization of mice with the myelin oligodendrocyte glycoprotein (MOG). Serum levels of MOG-specific antibodies and IFN-γ were also reduced in ARAP-deficient EAE mice compared to wild-type EAE mice. Moreover, adoptive transfer of ARAP-deficient T cells induced less severe EAE in recombination-activating gene 1-deficient mice than wild-type T cell transfer. These results strongly suggest that ARAP positively regulates T cell function, while ARAP deficiency in T cells reduces the severity and incidence of EAE.

## Introduction

T cells mediate cell-based immune responses in the adaptive immune system, protecting the host from infection while preventing self-reactivity. The interaction between a T cell and an antigen-presenting cell (APC) serves as the initiating event of the adaptive immune response. At the junction between a T cell and an APC, known as the immunological synapse (IS), T cells recognize antigens and initiate signaling events. A better understanding the initial stages of T cell signaling and the subsequent mechanisms of T cell activation provides valuable insights for developing novel prophylactic and therapeutic strategies in various diseases.

The process of T cell receptor (TCR) activation at the IS of T cells and APC has been investigated quite extensively (1–4). Previous studies have also demonstrated the roles of membrane micro-domains, also called membrane rafts, in initiating TCR signaling (5–9). Depending on the TCR stimulation, various signaling molecules concentrated within rafts induce actin polymerization and inside-out signaling to integrin activation at the T cell contact site (10–13). These F-actin- and integrin-dependent mechanisms increase adhesive IS formation (14–16).

Upon TCR stimulation, ZAP-70 translocates from the cytoplasm to the membrane rafts and phosphorylates signaling molecules that contain several tyrosine phosphorylation sites and protein-protein interaction domains (17–21). These signaling molecules then recruit other cytoplasmic signaling molecules to the rafts (17–21). Thus, we analyzed phosphotyrosine proteins that are recruited to membrane rafts following TCR activation (22). A novel adaptor protein, the sequence of which showed a similarity to a well-known T cell adaptor protein, adhesion and degranulation promoting adaptor protein (ADAP), was identified and named activation-dependent, raft-recruited ADAP-like phosphoprotein (ARAP) (22). Our previous study, which used *ARAP* gene-suppressed Jurkat T cells, showed that ARAP is required for proximal signaling, leading to T cell activation and integrin-mediated adhesion after TCR stimulation (22).

To investigate the role of ARAP in T cell biology *in vivo*, we generated mice with an *ARAP* gene deficiency. These mice are viable, breed normally, and undergo normal immune cell development. However, upon TCR activation, ARAP-deficient peripheral T cells exhibit defects in proximal signaling and inside-out activation of integrins, with decreased adhesion to ICAM-1 and fibronectin, as shown in ARAP-suppressed Jurkat T cells. ARAP-deficient T cells also inefficiently upregulated activation markers CD25 and CD69, produced less IL-2, and demonstrated reduced proliferation in response to TCR stimulation. In addition, ARAP-deficient mice presented less severe clinical scores in the myelin oligodendrocyte glycoprotein (MOG)-induced experimental autoimmune encephalomyelitis (EAE). The reduced severity and incidence of EAE were also shown in mice with a deficient *recombination activating gene (RAG)-1* gene after the adoptive transfer of ARAP-deficient T cells and wild-type (WT) B cells. These studies strongly suggest that ARAP is required for T cell function and consequently influences the clinical course of EAE in ARAP-deficient mice. Furthermore, these results indicate that ARAP plays a critical role in T cell biology, which may have important clinical implications.

## Results

### ARAP-deficient mice develop normally and present no overt phenotypic differences

**Figure 1:**
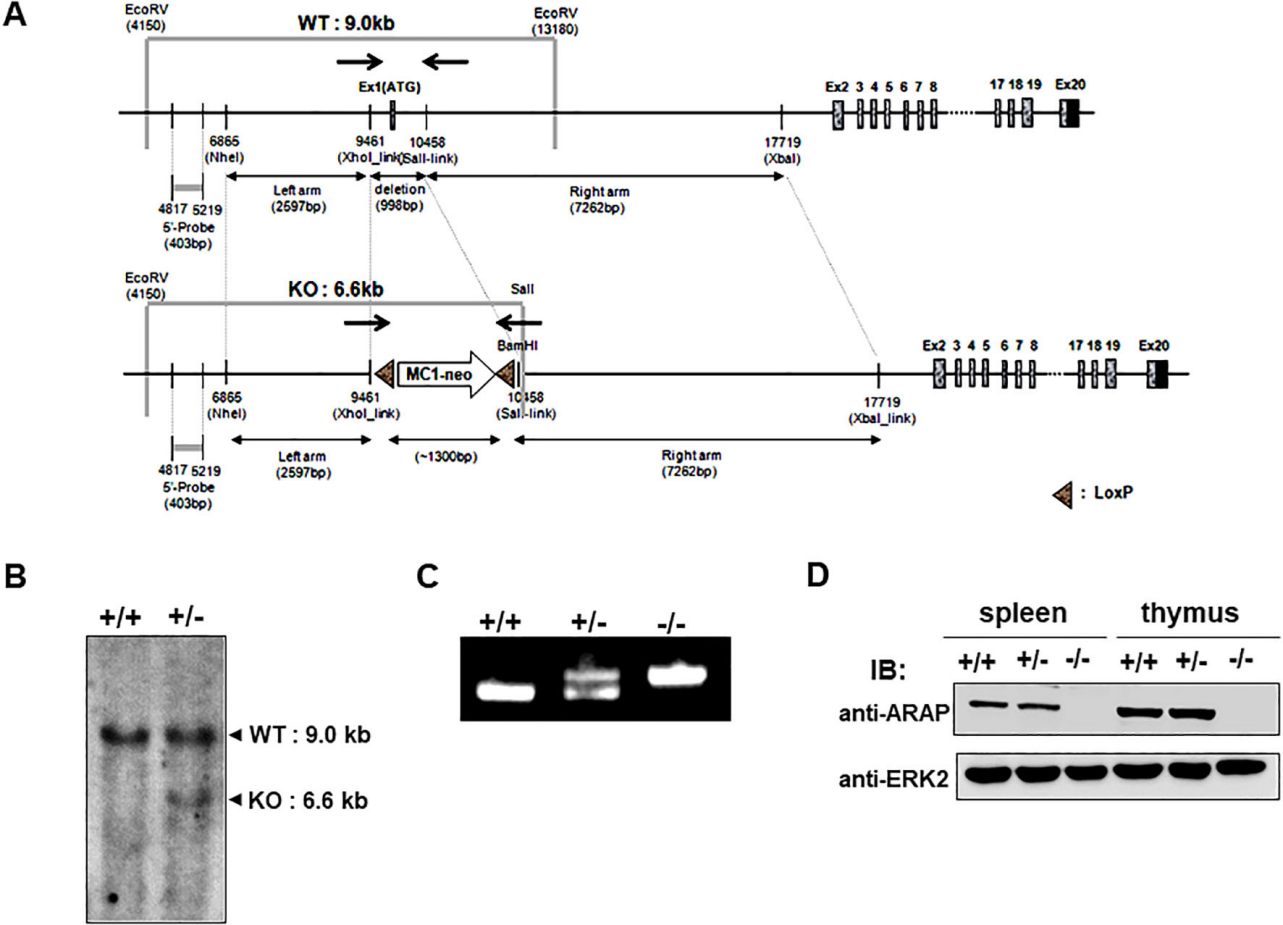
Schematic of the ARAP genomic locus and targeting strategy used to confirm the generation of an ARAP null allele. **(A)** The relative location of the coding exons within the WT ARAP locus (top) and the correctly targeted ARAP allele (bottom). Homologous recombination was employed to replace exon 1 in ARAP with a neomycin-resistant cassette. The region corresponding to the 403 bp genomic probe (5′ probe) used for the Southern blot analysis is also shown. The arrows indicate the location of the PCR primer sets for genotyping (→←). **(B)** Southern blot analysis of genomic DNA. Genomic DNA was isolated from ES cells, digested with EcoRV for WT (+/+) and EcoRV/SalI for the homologous recombinant (+/-), and separated using electrophoresis followed by transfer and hybridization with the 5′ probe. The expected genomic fragments after digestion are depicted in **(A)**. **(C)** PCR analysis for targeted locus. Genomic DNA was isolated from the tails of ARAP^+/+^, ARAP^+/-^, and ARAP^-/-^ mice. The primer sets indicated in **(A)** amplify a 998 bp product for WT and a ~1300 bp product containing sequences targeted for recombination. **(D)** IB analysis for ARAP expression. Total splenocytes and thymocytes were prepared from ARAP^+/+^, ARAP^+/-^, and ARAP^-/-^ mice. Cell lysates were resolved by electrophoresis followed by IB with a polyclonal rabbit anti-mouse ARAP antiserum. The anti-ERK2 blot is shown for the loading control.

To address the functional significance of ARAP *in vivo*, homologous recombination was performed to generate mice with an *ARAP* gene deficiency by replacing exon 1 (9461–10,458 bp (998 bp)) in the *ARAP* gene locus with a neomycin-resistance cassette (~1300 bp) (**Figure 1A**). *ARAP* gene targeting was confirmed by Southern blotting and PCR analysis of genomic DNA from WT (+/+), heterozygous (+/-), and *ARAP*-deficient (-/-) mice (**Figures 1B, C**). The ARAP protein expression was also compared in thymocytes and splenocytes isolated from ARAP +/+, +/-, and -/- mice (**Figure 1D**). Although ARAP is expressed in a broad range of tissues, as shown in our previous report (22), ARAP-deficient male and female mice were viable and fertile and developed normally, exhibiting no overt phenotypic differences.

**Figure 2:**
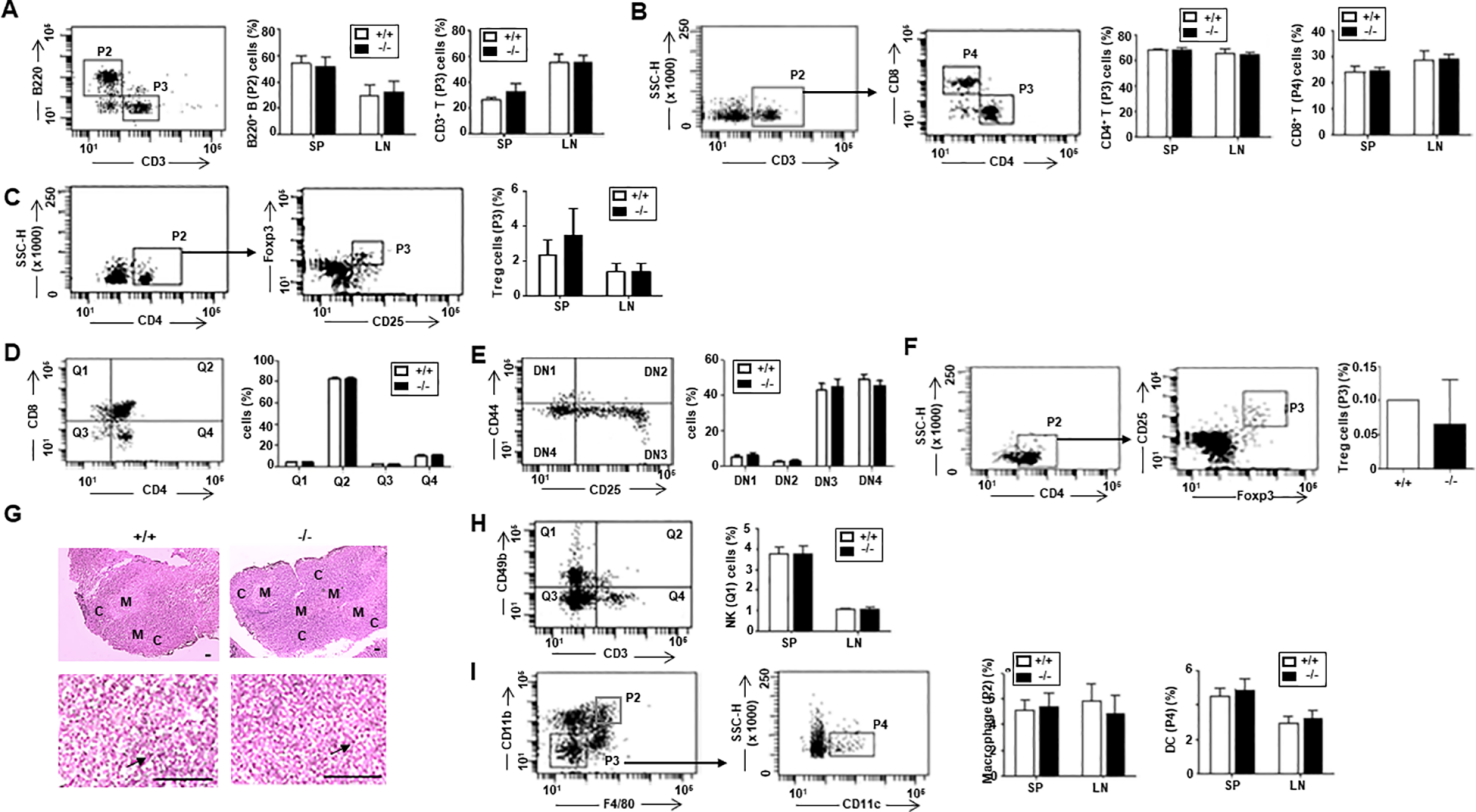
Normal immune cell population in ARAP-deficient mice. Total cells were obtained from the thymus, spleen (SP), and lymph node (LN) of 10- to 12-week-old WT (+/+) and ARAP-deficient (-/-) mice. Cells were surface stained with different color combinations of fluorochrome-conjugated Abs to the indicated proteins and subjected to flow cytometric analysis. Staining with isotype-matched, nonspecific, fluorochrome-conjugated control Abs was performed to establish background staining levels. Representative dot plots of fluorescence intensity and gating strategies are shown. The percentage of cells in each gate or quadrant is presented as the mean ± SEM of five separate experiments. Unpaired *t*-tests determined significant comparisons. **(A)** B (P2, B220^+^CD3^-^) and T (P3, CD3^+^B220^-^) cells. **(B)** CD3^+^CD4^+^ (P3) and CD3^+^CD8^+^ (P4) T cells. **(C)** Regulatory T cells (Tregs; P3, CD25^+^Foxp3^+^). **(D)** Thymic T cell precursors were analyzed for the expression of CD4 and CD8: CD4^-^CD8^+^ (Q1), CD4^+^CD8^+^ (Q2), CD4^-^CD8^-^ (Q3), and CD4^+^CD8^-^ (Q4). **(E)** CD4^-^CD8^-^ (Q3) double negative (DN) T cell precursors were analyzed for the expression of CD44 and CD25: CD25^-^CD44^+^ (DN1), CD25^+^CD44^+^ (DN2), CD25^+^CD44^-^ (DN3), and CD25^-^CD44^-^ (DN4). **(F)** Thymic Treg (P3, CD25^+^Foxp3^+^) cells. **(G)** Morphology of the thymus by hematoxylin/eosin staining. The upper panels show a low-power light micrograph of a thymic lobe, displaying the cortex (C) and medulla (M). The lower panels present a high-power light micrograph of the selected areas, with arrows indicating thymocytes. Scale bars = 100 μm. **(H)** NK (Q1, CD3^-^CD49b^+^) cells. **(I)** Macrophages (P2, F4/80^+^CD11b^+^) and DCs (P4, F4/80^-^CD11b^-^CD11c^+^).

No differences in the morphology (size and shape) of immune organs, including the spleen, lymph nodes, and thymus, were observed. Additionally, the distribution of B (B220^+^) and T (CD3^+^) cells, as well as T cell subsets (CD4^+^ or CD8^+^) and regulatory T cells (Treg), was normal in the spleen (SP) and lymph node (LN) of ARAP-deficient mice (**Figures 2A–C**). Analysis of T cell development in the thymus also revealed no abnormalities in the ARAP-deficient mice (**Figures 2D–F**). Additionally, the thymic structure in ARAP-deficient mice appeared normal (**Figure 2G**). Furthermore, populations of the natural killer cells (NK, CD49b^+^CD3^-^), macrophages (CD11b^+^F4/80^+^), and dendritic cells (DC, CD11c^+^CD11b^-^) were observed to be normal in the SP and LN of ARAP-deficient mice (**Figures 2H, I**). These results suggest that the immune system of ARAP-deficient mice is also likely to function normally.

### ARAP is required for proximal signaling and integrin-mediated adhesion following TCR stimulation

**Figure 3:**
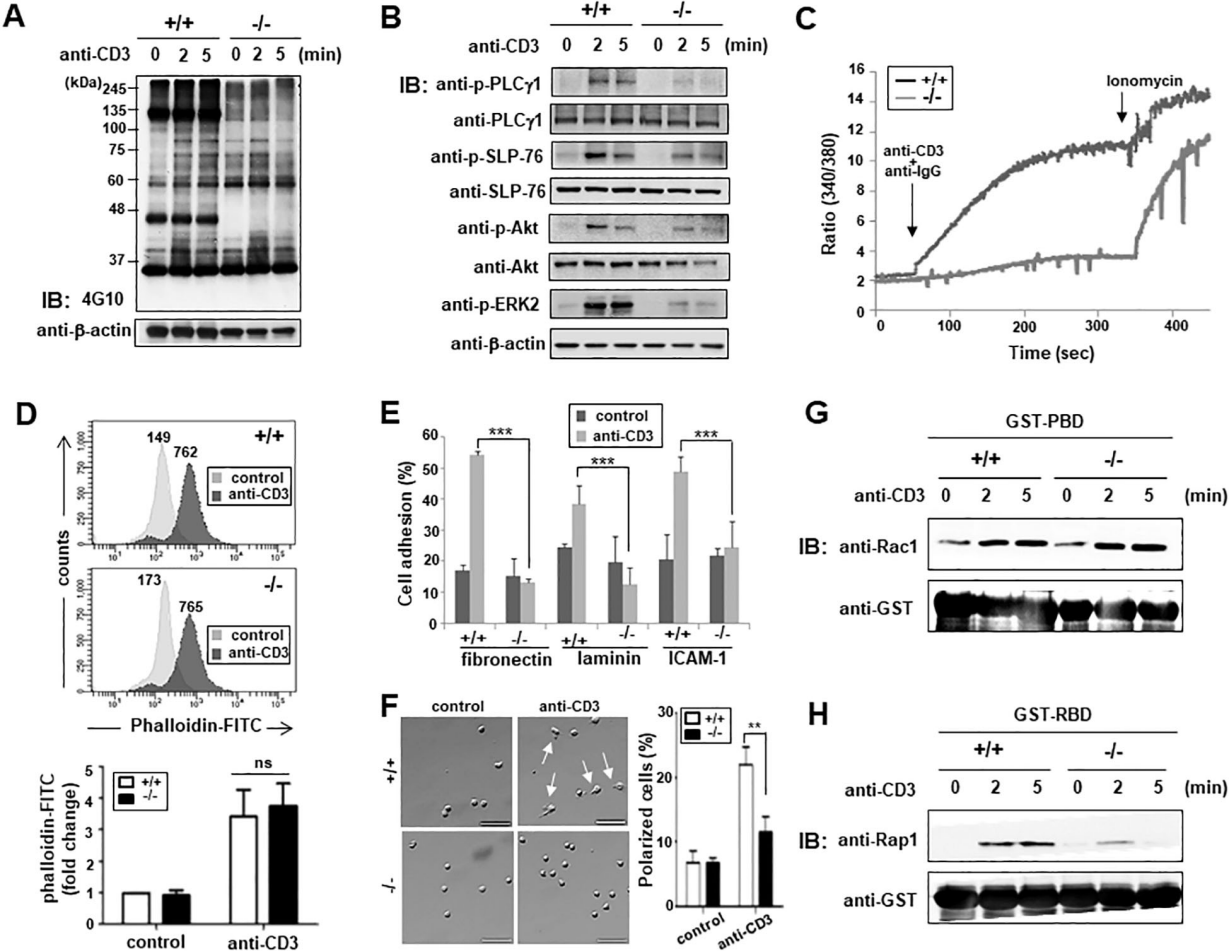
Impaired TCR proximal signaling and integrin-mediated T cell adhesion in ARAP-deficient mice. **(A, B)** Purified splenic T cells from WT (+/+) and ARAP-deficient (-/-) mice were stimulated using an anti-CD3 Ab followed by cross-linking at 37°C for the indicated durations. The lysates were resolved by SDS-PAGE, followed by IB analysis. Tyrosine-phosphorylation of total proteins **(A)** and total phosphorylation of PLCγ1, SLP-76, Akt, and ERK2 **(B)** are shown. The anti-β-actin blot is provided as the loading control. **(C)** Purified splenic T cells from WT (+/+) and ARAP-deficient (-/-) mice were loaded with Fura-2AM, and intracellular calcium elevation after TCR stimulation (anti-CD3 and anti-IgG) or ionomycin treatment was detected using a spectrofluorometer. **(D)** Purified splenic T cells from WT (+/+) and ARAP-deficient (-/-) mice were stimulated with anti-CD3 plus cross-linking Abs at 37°C for 30 min, stained with FITC-conjugated phalloidin, and assayed for cellular polymerized F-actin by flow cytometry. Numbers indicate the mean fluorescent intensities (MFIs) of cells in either the unstimulated control or stimulated samples. The histograms are representative of three separate experiments. Fold changes in the MFIs compared to unstimulated WT cells are demonstrated as the mean ± SEM of three separate experiments. Unpaired *t*-tests determined significant comparisons; ns indicates no significance. **(E)** Purified splenic T cells from WT (+/+) and ARAP-deficient (-/-) mice were either left unstimulated (control) or stimulated using anti-CD3 plus goat anti-hamster Abs at 37°C for 30 min. These cells were then added to 96-well plates coated with fibronectin, laminin, or anti-mouse ICAM-1 Abs. Adherent cells were obtained and counted after incubating at 37°C for 30 min. The percentage of adherent cells was calculated. The data are the mean ± SD of three independent experiments performed in triplicate. Statistical significance was analyzed using a two-way ANOVA with Bonferroni multiple comparison tests. ^***^ *p* < 0.001. **(F)** Purified splenic T cells from WT (+/+) and ARAP-deficient (-/-) mice were either left unstimulated (control) or stimulated using anti-CD3 plus goat anti-hamster Abs at 37°C for 30 min. These cells were plated on fibronectin-coated coverslips, incubated at 37°C for 30 min, fixed, and analyzed by microscopy. Representative images are shown, with arrows indicating polarized T cells displaying a clear uropod. Scale bars = 50 μm. The percentage of polarized cells is presented as the mean ± SD from four independent experiments (total: 555 cells for WT (+/+) and 533 cells for ARAP-deficient (-/-) mice). Statistical significance was determined using unpaired *t*-tests. ^**^ *p* < 0.01. **(G, H)** Splenic T cells purified from WT (+/+) and ARAP-deficient (-/-) mice were stimulated with anti-CD3 plus goat anti-hamster Abs at 37°C for the indicated durations. Cells were lysed, and the lysates were incubated with GST-PBD fusion protein beads **(G)** or GST-RBD fusion protein beads **(H)** for 10 min. The proteins that were pulled down by the beads were resolved by SDS-PAGE followed by IB for active GTPase, Rac1 **(G)**, or Rap1 **(H)**. IB also showed equal amounts of used fusion proteins with anti-GST Ab. Data are representative of three independent experiments.

Previous studies using ARAP-suppressed Jurkat T cells discovered defects in TCR signaling (22). Therefore, we investigated whether similar defects were observed in T cells from ARAP-deficient mice. Here, reduced tyrosine phosphorylation of proteins was found in T cells isolated from mice lacking ARAP following TCR stimulation compared with T cells from WT mice (**Figure 3A**). Phosphorylation of individual proteins involved in the proximal signaling pathways following TCR stimulation was examined by immunoblotting using an anti-phosphoprotein antibody (Ab). Following TCR stimulation, PLCγ1, SLP-76, Akt, and ERK phosphorylation were reduced in ARAP-deficient T cells (**Figure 3B**). An increase in the cytosolic Ca^2+^ observed following phospho-PLCγ1 activation was also reduced in ARAP-deficient T cells (**Figure 3C**).

We also found defects in T cell adhesion, but not actin polymerization, after TCR stimulation in ARAP-deficient mouse T cells, similar to that observed in ARAP-suppressed Jurkat T cells (22). TCR-induced F-actin formation between WT and ARAP-deficient T cells was compared by analyzing the fluorescence emitted from the FITC-conjugated phalloidin (**Figure 3D**). ARAP deficiency did not influence actin polymerization following TCR stimulation in T cells (**Figure 3D**). In contrast, TCR-mediated integrin activation was affected by ARAP deficiency. T cell adhesion to matrix proteins such as fibronectin and laminin, and ICAM-1 after TCR stimulation was significantly reduced in ARAP-deficient T cells (**Figure 3E**). T cell polarization induced by fibronectin binding to integrin was also diminished in TCR-stimulated ARAP-deficient T cells (**Figure 3F**). In agreement with these results, comparable activation kinetics were observed for Rac1 GTPases in WT and ARAP-deficient T cells following TCR stimulation (**Figure 3G**); however, TCR-induced Rap1 GTPase activity was inhibited in ARAP-deficient T cells (**Figure 3H**).

### ARAP is required for T cell activation and cytokine secretion following TCR stimulation

**Figure 4:**
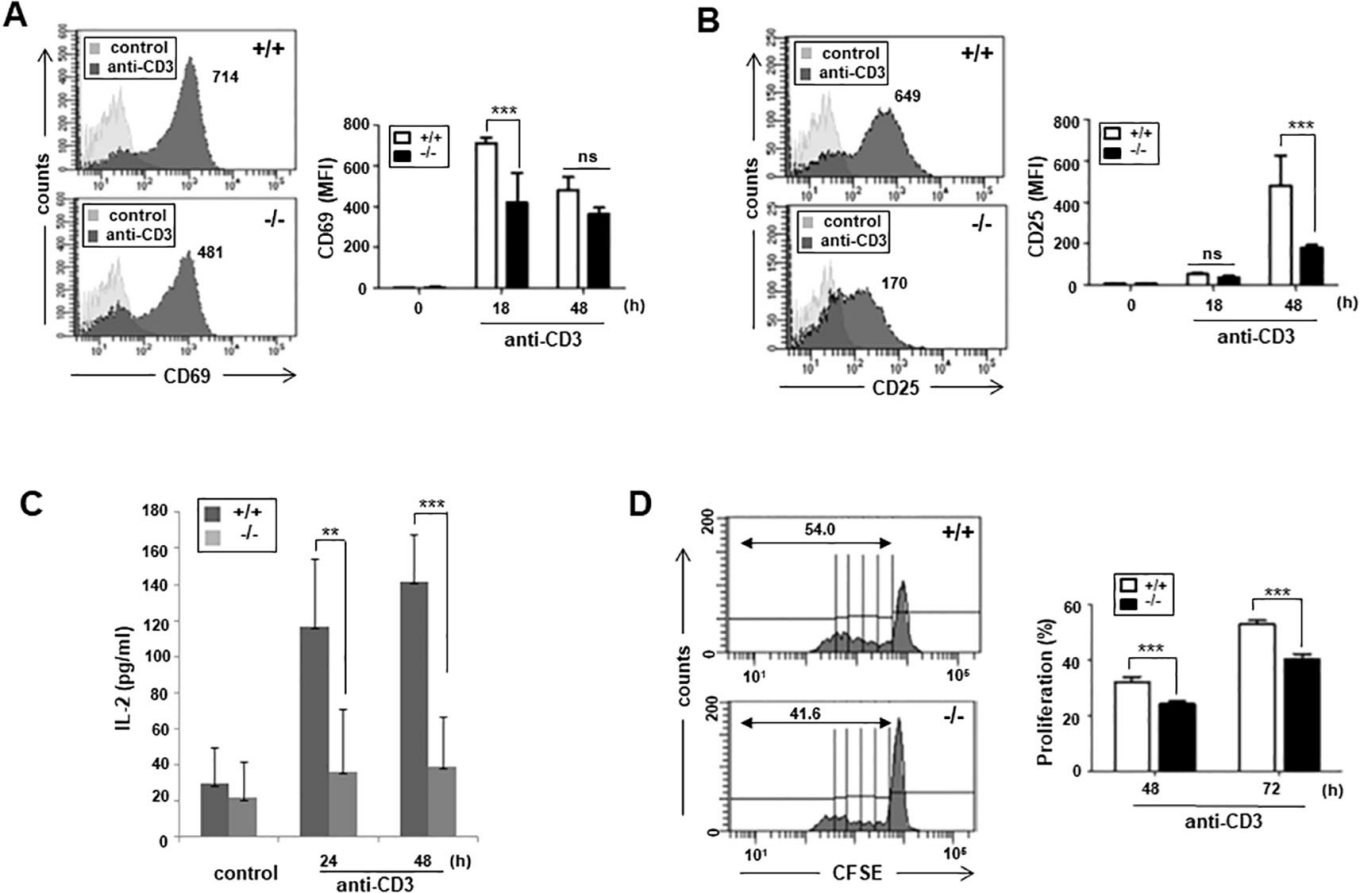
Impaired T cell activation, proliferation, and IL-2 secretion in ARAP-deficient mice. **(A, B)** Purified splenic T cells from WT (+/+) and ARAP-deficient (-/-) mice were cultured with plate-bound anti-CD3 Abs for 18 or 48 h, stained with anti-CD69 **(A)** or anti-CD25 Ab **(B)**, and analyzed by flow cytometry. Numbers indicate the MFIs of cells expressing CD69 **(A)** or CD25 **(B)** after stimulation. The histograms are representative of three separate experiments. The data are the mean ± SEM of three independent experiments. Statistical significance was analyzed using a two-way ANOVA with Bonferroni multiple comparison tests. ^***^ *p* < 0.001. **(C)** Purified splenic T cells from WT (+/+) and ARAP-deficient (-/-) mice were cultured on plates coated with anti-CD3 Abs at 37°C for the indicated durations. ELISA was used to measure the IL-2 production in the supernatants. The data are presented as the mean ± SEM of three independent experiments. Statistical significance was analyzed using a two-way ANOVA with Bonferroni multiple comparison tests. ^**^ *p* < 0.01, ^***^ *p* < 0.001. **(D)** WT (+/+) and ARAP-deficient (-/-) splenic T cells were labeled with CFSE and cultured on the plates coated with anti-CD3 Abs at 37°C for 48 or 72 h. Proliferation of CFSE-labeled T cells was analyzed using flow cytometry. Representative histograms after stimulation for 72 h are shown, and the numbers indicate the percentage of cells with diluted fluorescence after division. The data are the mean ± SEM of triplicate experiments, with similar results obtained more than three times. Statistical significance was analyzed using a two-way ANOVA with Bonferroni multiple comparison tests. ^***^ *p* < 0.001.

Next, we addressed whether impaired proximal TCR signaling in ARAP-deficient T cells affects T cell activation and function. The expression of activation markers was monitored following TCR stimulation using surface fluorescence staining and flow cytometry. The early activation marker, CD69, levels after 18 h TCR stimulation were significantly reduced in ARAP-deficient T cells compared to WT T cells (**Figure 4A**). The late activation marker, CD25, expression was also significantly reduced in ARAP-deficient T cells after TCR stimulation for 48 h (**Figure 4B**). Furthermore, TCR stimulation significantly reduced the IL-2 cytokine production in ARAP-deficient T cells compared to WT T cells (**Figure 4C**). Consequently, TCR-induced cell proliferation, measured using flow cytometry by detecting CFSE dye dilution, was less efficient in ARAP-deficient T cells than in WT T cells (**Figure 4D**).

### ARAP-deficient mice exhibit less severe experimentally induced autoimmunity

**Figure 5:**
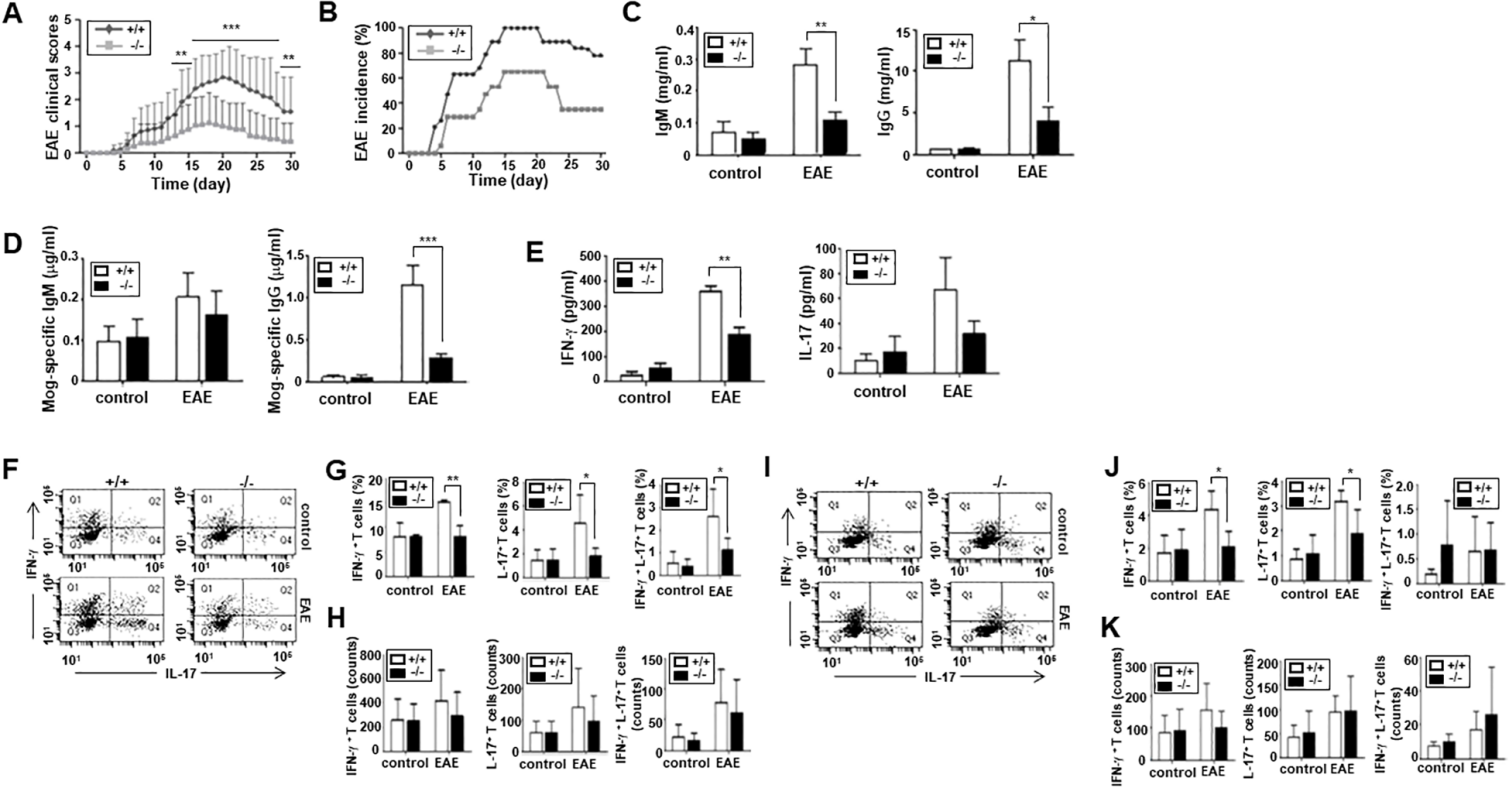
Reduced severity and incidence of EAE in ARAP-deficient mice. **(A, B)** WT (+/+) and ARAP-deficient (-/-) mice were immunized with MOG peptide 35–55 (200 μg) emulsified with complete Freund’s adjuvant, as described in the Materials and Methods. The severity of EAE **(A)** is presented as mean clinical scores. Data are pooled from four independent experiments and presented as the mean ± SEM (n = 16). Statistical significance was analyzed using an unpaired *t*-test. ^**^ *p* < 0.01, ^***^ *p* < 0.001. The incidence of EAE **(B)** is shown by the percentage of mice that developed the disease. **(C, D)** Anti-IgM, anti-IgG, and anti-MOG-specific Abs in sera were collected on day 15 after an ELISA-measured immunization. The data are the mean ± SEM of four independent experiments performed in triplicate. Unpaired *t*-tests analyzed statistical significance. ^*^ *p* < 0.05, ^**^ *p* < 0.01, ^***^ *p* < 0.001. **(E)** ELISA was used to measure the IFN-γ and IL-17 cytokine levels in sera collected on day 15 after immunization. The data are the mean ± SEM of four independent experiments performed in triplicate. Unpaired *t*-tests analyzed statistical significance. ^**^ *p* < 0.01. **(F–K)** CD4^+^ T cells were isolated from the spleen **(F–H)** or draining lymph nodes **(I–K)** of WT (+/+) and ARAP-deficient (-/-) mice 15–18 days after immunization. Cells were stained for intracellular cytokines and surface proteins using various fluorochrome-conjugated Ab combinations and analyzed by flow cytometry, as shown in **Figure 2** **(F, I)**. The percentages of IFN-γ<συπ>+</sup> T cells (Q1), IL17^+^ T cells (Q4), and IFN-γ<συπ>+</sup> IL-17^+^ double-positive T cells (Q2) are presented as the mean ± SEM from three independent experiments for spleen cells **(G)** and four independent experiments for draining lymph node cells **(J)**. Statistical significance was determined using unpaired *t*-tests (^**^ *p* < 0.01, ^*^ *p* < 0.05). The absolute cell numbers in Q1, Q2, and Q4 were obtained from a total of 10,000 cells analyzed by flow cytometry. The absolute cell counts are presented as the mean ± SEM from three independent experiments for spleen cells **(H)** and four independent experiments for draining lymph node cells **(K)**. Statistical significance was determined using unpaired *t*-tests.

Next, we examined whether an ARAP deficiency affects the immune functions of mice *in vivo*. We adopted an EAE model; both WT and ARAP-deficient mice were immunized with the MOG peptide, and the clinical course of EAE was investigated. A significant reduction was observed in the maximum disease scores, and complete remission appeared in the ARAP-deficient mice compared to WT mice (**Figure 5A**). The EAE incidence was also delayed in ARAP-deficient mice; meanwhile, the incidence rate during EAE was reduced to almost half of that observed in WT mice (**Figure 5B**). The IgM and IgG levels were significantly reduced in the sera obtained from EAE-induced ARAP-deficient mice compared to WT mice (**Figure 5C**), with the MOG-specific IgG isotype significantly reduced on day 15 post-immunization (**Figure 5D**). The level of serum cytokines, IFN-γ and IL-17, was also reduced in the EAE-induced ARAP-deficient mice on day 15 post-immunization compared to the WT mice (**Figure 5E**). These results corresponded well with the analysis of T cell subsets in the spleen (**Figures 5F–H**) and draining lymph nodes (**Figures 5I–K**) on day 15 post-immunization. A reduction in the population of IFN-γ-secreting-Th1 and IL-17-secreting-Th17 cells was observed in EAE-induced ARAP-deficient mice compared to WT mice. However, there was no significant difference in the absolute counts of each cytokine-secreting cell types (**Figures 5H, K**). A decrease in the Th2 or Treg subset population was also observed in the ARAP-deficient mice; however, no significant difference was shown between the WT and ARAP-deficient mice (data not shown).

**Figure 6:**
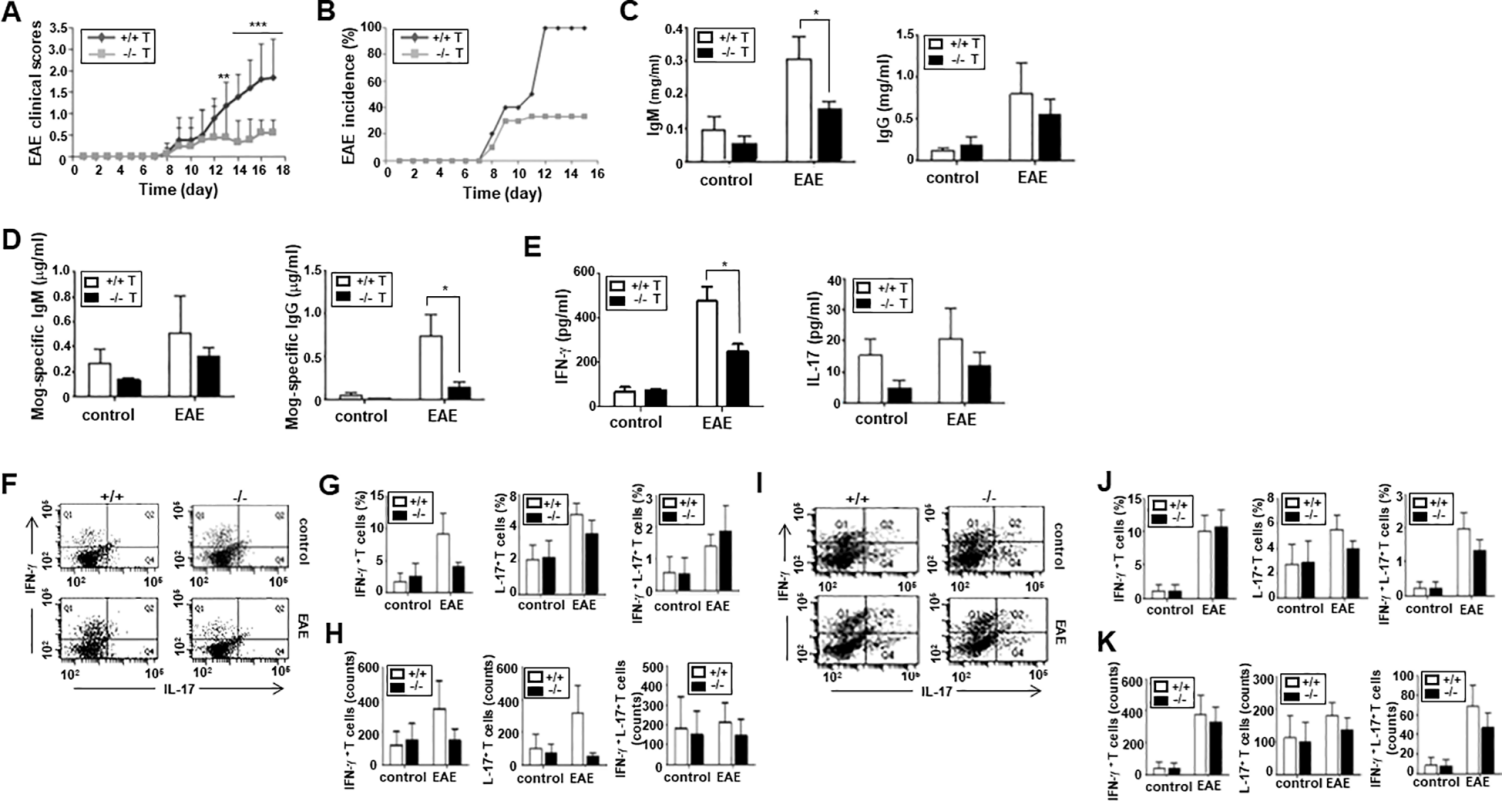
Severity and incidence of EAE in Rag1-deficient mice adoptively transferred with lymphocytes. Rag1-deficient mice were reconstituted with T cells from WT (+/+) or ARAP-deficient (-/-) mice and B cells from WT mice. EAE was induced in these mice, as in **Figure 5**. **(A)** The clinical scores are pooled from three independent experiments (mean ± SEM, n = 13). Unpaired *t*-tests were used to determine statistical significance. ^**^ *p* < 0.01, ^***^ *p* < 0.001. **(B)** The incidence of EAE is shown by the percentage of mice that developed the disease in a group. **(C, D)** ELISA was used to measure the anti-IgM, anti-IgG, anti-MOG-specific Ab levels in sera collected on day 15 after immunization. The data are presented as the mean ± SEM of three independent experiments performed in triplicate. Unpaired *t*-tests analyzed statistical significance. ^*^ *p* < 0.05. **(E)** ELISA was used to measure the IFN-γ and IL-17 cytokine levels in sera collected on day 15 after immunization. The data are the mean ± SEM of three independent experiments performed in triplicate. Unpaired *t*-tests analyzed statistical significance. ^*^ *p* < 0.05. **(F–K)** CD4^+^ T cells were isolated from the spleen **(F–H)** or draining lymph nodes **(I–K)** of Rag1-deficient mice reconstituted with T cells from WT (+/+) or ARAP-deficient (-/-) mice and B cells from WT mice 15–18 days after immunization. Cytokine-secreting cells were analyzed as shown in **Figure 5**. The percentages and absolute counts of cytokine-secreting cells are presented as the mean ± SEM from three independent experiments. Statistical significance was determined using unpaired *t*-tests.

As previously presented, ARAP is expressed broadly in various tissues and cells (22). Thus, decreased clinical score severity in the MOG-induced EAE of ARAP-deficient mice could involve possible functional defects in many cell types. To further demonstrate that impaired T cell function is responsible for the reduced EAE scores in ARAP-deficient mice, T cells from either WT or ARAP-deficient mice were transferred alongside B cells from the WT control mice to Rag1-deficient mice. EAE was induced in these Rag1-deficient mice adaptively transferred with lymphocytes using MOG peptides. As shown in the WT and ARAP-deficient mice (**Figures 5A, B**), the clinical scores and incidence of EAE were significantly reduced in the mice receiving the ARAP-deficient T cells compared with those receiving the WT T cells (**Figures 6A, B**). The IgM and IgG levels were reduced in the sera obtained from the EAE-induced mice that received the ARAP-deficient T cells; the MOG-specific IgG isotype was also shown to be significantly decreased on day 15 post-immunization in mice with ARAP-deficient T cells (**Figures 6C, D**). Moreover, the serum level of IFN-γ rather than IL-17 was significantly reduced in EAE-induced Rag1-deficient mice with ARAP-deficient T cells on day 15 post-immunization (**Figure 6E**). The T cell subset population was also analyzed in the spleen (**Figures 6F–H**) and draining lymph nodes (**Figures 6I–K**) of Rag1-deficient mice that were adaptively transferred with lymphocytes on day 15 post-immunization. A reduction in both the population and the absolute counts of IFN-γ-secreting-Th1 cells was observed in the spleen of the mice receiving the ARAP-deficient T cells compared to those receiving WT T cells, although this difference was not statistically significant. Overall, no significant differences were observed in the T cell subset population or in the absolute counts of cytokine-secreting cells in EAE-induced Rag1-deficient mice receiving either WT or ARAP-deficient T cells. These results strongly suggest that the function of IFN-γ secreting T cells is impaired in ARAP-deficient mice, contributing to the reduced severity of EAE.

## Discussion

ARAP is a phosphoprotein that is recruited to the TCR signaling complex formed at the IS after TCR stimulation (22). ARAP contains a proline-rich domain at the N-terminus, a SH3 domain at the C-terminus, and several tyrosine motifs between these domains (22). ARAP shares sequence homology with the T cell adaptor protein ADAP. However, the sequences for the two putative nuclear localization sites, an internal SH3 domain, and a binding site for an enabled/vasodilator-stimulated phosphoprotein homology domain are absent in ARAP (22–26).

Both ARAP and ADAP are cytosolic adaptor proteins that facilitate signal transduction from cell surface receptors by assembling critical effector molecules. Upon TCR stimulation, both ARAP and ADAP are tyrosine-phosphorylated and associate with another cytosolic adaptor, SLP-76, through its SH2 domain (22, 27, 28). As shown in SLP-76-deficient T cells (29, 30), ARAP-deficient T cells similarly uncoupled TCR-stimulated protein tyrosine kinases from downstream activation signals, including TCR-induced phosphorylation of SLP-76 and PLCγ-1, followed by MAPK activation and calcium elevation (**Figures 3A–C**). Unlike the SLP-76 or ARAP deficiency, T cells with an ADAP deficiency did not exhibit defects in early biochemical events following TCR stimulation (31, 32). As shown in the ADAP-deficient T cells (33, 34), an ARAP deficiency also resulted in defective integrin-mediated adhesion after TCR stimulation (**Figures 3E, F, H**). However, an ARAP deficiency in T cells did not affect actin polymerization triggered by TCR stimulation (**Figures 3D, G**), compared to the SLP-76 and ADAP deficiencies (35–37). Although some contrasting signaling events exist in ARAP- or ADAP-deficient T cells following TCR stimulation, the long-term activation of T cells, such as the upregulation of the surface antigens, proliferation, and IL-2 production, were defective in ARAP- and ADAP-deficient T cells (**Figure 4**) (31, 32).

ARAP is widely expressed in hematopoietic and non-hematopoietic cells (22), whereas ADAP is expressed in T cells, myeloid cells, and platelets (25, 26). Although ARAP is expressed ubiquitously, mice lacking the *ARAP* gene were viable and fertile and showed no obvious abnormalities. Contrastingly, defective thymocyte development and selection were previously reported in ADAP-deficient mice (38, 39). However, an ARAP deficiency did not affect overall immune cell development (**Figure 2**).

Previous studies also reported that ADAP deficiency can affect autoimmune diseases both positively and negatively (40, 41). An enhanced incidence of autoimmune diabetes was reported in ADAP-deficient, diabetes-prone TCR transgenic mice, which could result from ineffective thymic T cell homeostasis (40). The attenuated course of EAE was also noticed in ADAP-deficient mice; however, this mild EAE was not due to an intrinsic defect of T cell activation but rather from T cell-independent mechanisms (41).

This study demonstrates that ARAP-deficient mice developed a milder clinical course and lower incidence upon active EAE induction by immunization with MOG peptide (**Figures 5A, B**). The impaired TCR-mediated proximal signaling and activation in ARAP-deficient T cells (**Figures 3**, **4**) could explain the reduced EAE severity shown in ARAP-deficient mice. Mice with the deficient ARAP did not demonstrate altered overall immune cell homeostasis, including the B cell population (**Figure 2**). Thus, the low level of Ab production in the context of EAE (**Figures 5C, D**) could result from a decrease in T cell-mediated help in ARAP-deficient mice. Moreover, analyses of the immune cell population, including T cells in the spleen and draining lymph nodes of both WT and ARAP-deficient mice on day 15 post-immunization, showed no significant difference (data not shown). Although TCR-mediated proliferation was reduced in ARAP-deficient T cells (**Figure 4D**), the total T cell population was not significantly different in EAE-induced WT and ARAP-deficient mice. These could be explained by the secondary effects of cytokines and other factors produced by long-term activation during EAE.

EAE is a CD4^+^ T cell-mediated disease model of multiple sclerosis, and the generation of effector CD4^+^ T cells is a critical event in the progression of EAE (42). Further analyses of CD4^+^ T cell subsets in the spleen and draining lymph nodes of EAE-induced mice showed that the ARAP-deficient mice exhibited a reduction in the subset population of Th1, Th17, and Th1-like Th17 (**Figures 5F, G, I, J**). However, there was no significant difference in the absolute counts of each cytokine-secreting cell types (**Figures 5H, K**). Moreover, Rag1-deficient mice alleviated the clinical course and incidence of EAE after the adoptive transfer of ARAP-deficient T cells (**Figures 6A, B**). ARAP-deficient T cells in Rag1-deficient mice also produced less Ab and IFN-γ in the context of EAE (**Figures 6C–E**). However, no significant differences were observed in the T cell subset population or in the absolute counts of cytokine-secreting cells in EAE-induced Rag1-deficient mice receiving either WT or ARAP-deficient T cells. These data suggest that reduced cytokine secretion, particularly IFN-γ, rather than changes in the absolute cell number, is likely the primary factor contributing to the decreased severity of EAE in ARAP-deficient mice. Furthermore, these results further indicate that the reduction in disease severity in the absence of ARAP is due to intrinsic functional defects in T cells.

This study investigated the physiological role of ARAP in ARAP-deficient mice. In marked contrast to ADAP, despite sequence and functional similarities, ARAP is not involved in the development of thymocytes but plays a role in coupling TCR stimulation to proximal activation signaling. ARAP-deficient mice display an attenuated clinical course and incidence of EAE due to a defect in T cell function. These results indicate that ARAP plays a critical role in T cell biology that ADAP cannot replicate.

**Figure 7:**
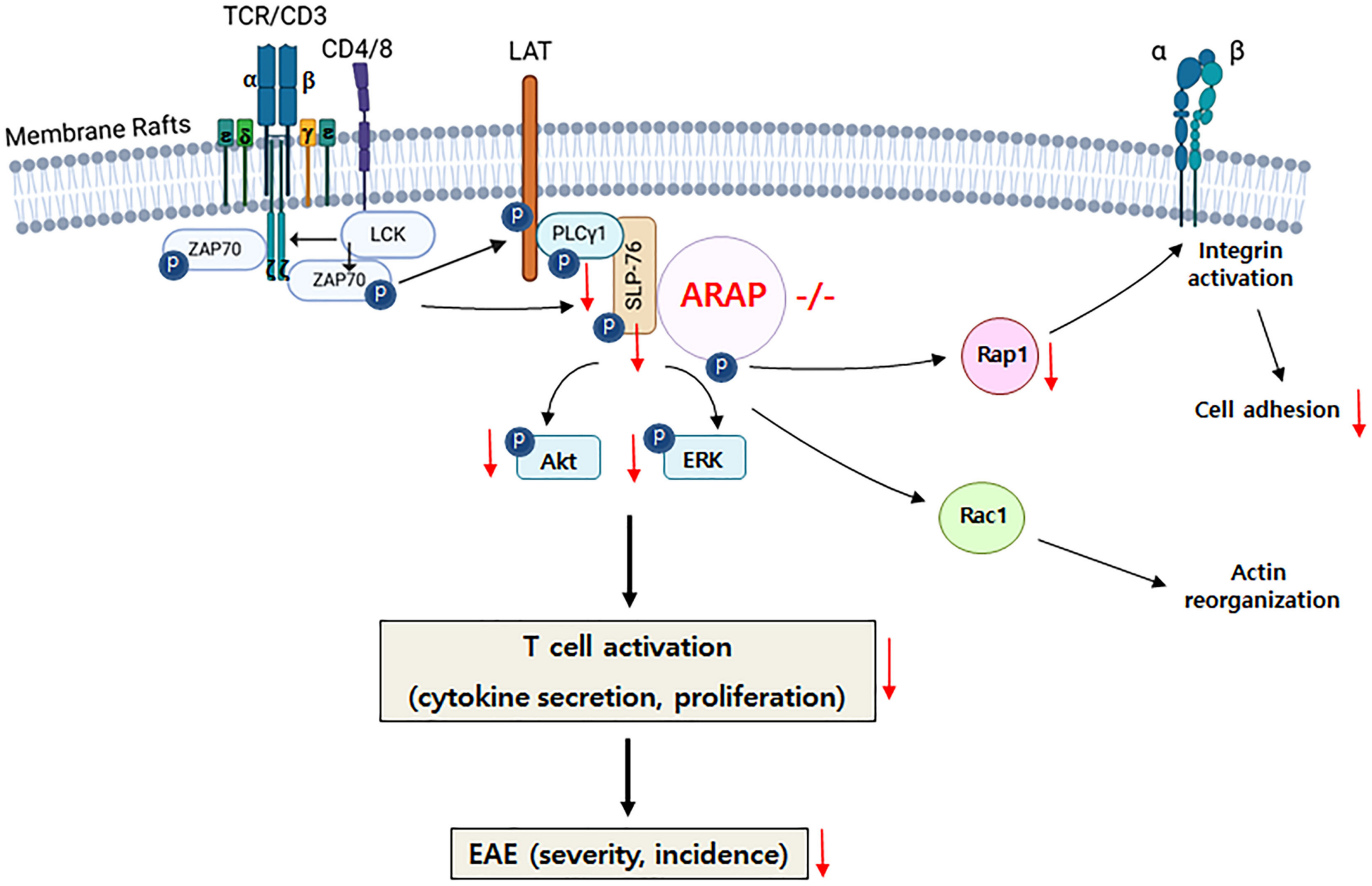
Graphical summary of ARAP’s role in T cell biology. In the absence of ARAP, proximal signaling through the TCR, including phosphorylation and activation of PLC-γ1, SLP-76, Akt, and ERK, as well as integrin-mediated adhesion, is inhibited. The impaired signaling in ARAP-deficient T cells leads to reduced activation, proliferation, and cytokine secretion. Consequently, EAE-induced ARAP-deficient mice exhibit lower cytokine secretion, particularly IFN-γ, and less severe disease scores.

The key findings on the role of ARAP in TCR signaling and the induction of EAE are graphically summarized in **Figure 7**.

## Materials and methods

### Generation of ARAP-deficient (-/-) mice

Homologous recombination was performed using a targeting vector that replaced a part of the ARAP exon 1 locus with a neomycin-resistance cassette (Macrogen Inc., Seoul, South Korea). The targeting construct was electroporated into 129 strain embryonic stem cells, and targeted clones were identified by Southern blotting. The targeted cells were microinjected into C57BL/6 blastocysts. The resulting male chimeras were crossed with female C57BL/6 mice to generate F1 ARAP heterozygous (+/-) germline mice. Heterozygous mice were intercrossed to produce F2 mice, which were further backcrossed with the C57BL/6 strain for 10 generations. DNA genotyping was performed from tail and toe cuttings by PCR using the 5′-gatcagctcaggcaggaaac-3′ and 5′-tagcttttcaacaccccagc-3′ primers. DNA fragments were generated for WT (1335 bp) and knock-out (1637 bp) samples. Per the institutional guidelines, mice were bred and maintained under specific pathogen-free conditions at the Ewha Laboratory Animal Genomic Center animal facility. Mice aged 8 to 12 weeks were used for all experiments.

### Antibodies and reagents

Anti-mouse CD3ε (145-2C11) monoclonal antibody (mAb), purchased from BD PharMingen (San Diego, CA, USA), was used for cell stimulation. For immunoblotting (IB), we used anti-PLCγ1 and anti-phosphotyrosine (4G10) mAbs (Upstate Biotechnology Inc., Lake Placid, NY, USA), in addition to anti-SLP-76, anti-Akt, anti-Erk2, anti-Rac1, anti-Rap1, anti-GST, and anti-phospho-PLCγ1 (p-PLCγ1, Y783) polyclonal Abs (Santa Cruz Biotechnology Inc., Santa Cruz, CA, USA), and anti-phospho-Akt1 (p-Akt, S473), anti-phospho-ERK1/2 (p-ERK, T202/Y204), and anti-phospho-SLP-76 (p-SLP-76) mAbs (Cell Signaling Technology Inc., Danvers, MA, USA). We generated a rabbit polyclonal anti-ARAP Ab using purified glutathione-S-transferase (GST)-conjugated ARAP-C-terminus (ARAP-CT, aa 546–728) as the immunogen (Peptron Inc., Daejeon, South Korea). Anti-mouse IgG and anti-rabbit horseradish peroxidase (HRP)-conjugated IgG secondary Abs were acquired from Bio-Rad (Hercules, CA, USA); anti-Armenian hamster IgG from Jackson ImmunoResearch Laboratories Inc. (West Grove, PA, USA). For the flow cytometry analysis, we used peridinin chlorophyll protein (PerCP)-conjugated rat anti-mouse CD4 (RM4-5), fluorescein isothiocyanate (FITC)-conjugated rat anti-mouse CD8a (53-6.7), FITC-conjugated rat anti-mouse CD45R/B220 (RA2-6B2), phycoerythrin (PE)- or allophycocyanin (APC)-conjugated anti-CD3ε (145-2C11) mAbs from BD Pharmingen, and biotin-conjugated anti-CD11b (Mac-1), anti-CD49b (pan-NK cells, DX5), anti-CD25, anti-CD44, FITC-conjugated IFN-γ, PE-conjugated anti-CD69, anti-IL-4, anti-IL-17a, anti-foxp3 Abs from eBioscience (San Diego, CA, USA), and FITC-conjugated streptavidin from Jackson ImmunoResearch Laboratories Inc. (West Grove, PA, USA). Abs used for IgM and IgG ELISA were obtained from SouthernBiotech (Birmingham, AL, USA). Enhanced chemiluminescence (ECL) reagent was purchased from Amersham Pharmacia Biotech Co. (Arlington Heights, IL, USA). Fura-2 acetoxy-methyl ester (Fura-2 AM) and CellTrace™ CFSE were purchased from ThermoFisher Scientific Inc. (Waltham, MA, USA). FITC-phalloidin, laminin, bovine serum albumin (BSA), paraformaldehyde (PFA), Harris hematoxylin, eosin Y, poly-L-lysine, and complete Freund’s adjuvant were obtained from Sigma-Aldrich Inc. (St Louis, MO, USA). The Tissue-Tek^®^ optimal cutting temperature (OCT) compound was purchased from Sakura Finetechnical Co., Ltd. (Tokyo, Japan). Heat-killed *Mycobacterium tuberculosis* was from DIFCO Laboratories (Detroit, MI, USA) and pertussis toxin was from List Biological Laboratories (Campbell, CA, USA). Human fibronectin, GolgiStop™ (monensin), Cytofix/Cytoperm™ reagent from BD Biosciences (San Jose, CA, USA), ICAM-1-Fc chimera from R&D Systems Inc. (Minneapolis, MN, USA), magnetic particle-labeled streptavidin, pan- or CD4^+^-T cell isolation kit from Miltenyi Biotech Inc. (Auburn, CA, USA), and mouse IL-2, IFN-γ, IL-17 Ready-SET-Go ELISA sets from eBioscience were also used. MOG p35–55 corresponding to a mouse peptide sequence was obtained from Peptron Inc.

### Purification and activation of T cells

Splenic cell suspensions were prepared, and red blood cells were removed using hypotonic lysis. The T cells were enriched using a pan-T cell isolation kit, according to the manufacturer’s instructions. More than 96% of the resulting cells were CD3^+^ T cells. Purified mouse splenic T cells were rested in the Gibco™ RPMI1640 medium (ThermoFisher Scientific) at 5 × 10^7^ cells/mL and incubated with either control or anti-mouse CD3ε Abs (1 μg/mL) followed by cross-linking with a secondary Ab at 37°C for the indicated times.

### Induction and clinical evaluation of EAE

Purified MOG peptide (p35–55) synthesized corresponding to the mouse sequence, MEVGWYRSPFSRVVHLYRNGK, was obtained (Peptron Inc., Daejeon, South Korea). EAE was induced according to the procedures described previously (43). Sex-matched 8- to 12-week-old mice were immunized by administering 200 μg MOG p35–55 emulsified in complete Freund’s adjuvant containing 800 μg of heat-killed *Mycobacterium tuberculosis* subcutaneously. Intraperitoneal injection with 200 ng of pertussis toxin was followed on days 0 and 2. Mice were monitored daily for signs of disease and graded on a scale of increasing severity from 0 to 5, as described previously (44, 45). Daily clinical scores were calculated as the average of all individual disease scores for each group. No sex preference was observed for EAE induction, and both sexes were used in the experiments. However, sex was matched within each experiment. For adoptive transfer experiments, B cells were purified from the spleens of WT mice as previously described (46), and T cells were purified from the spleens or lymph nodes as described above in the “Purification and activation of T cells” section. The purity of B and T cells was determined by flow cytometry before adoptive transfer. Both B220^+^ B cells and CD3^+^ T cells had a purity of more than 96% (**Supplementary Figure 1**). Equal amounts of purified B and T cells (2 × 10^7^) were mixed and transferred into Rag1-deficient mice by tail vein injection. Twenty-four hours after the transfer, recipient mice were immunized to induce EAE. Ewha Institutional Animal Care and Use Committee approved all mouse protocols.

### Thymus tissue staining

Histochemistry was performed as previously described (47, 48). The thymus was removed and fixed in 4% PFA for 1h. The tissues were dehydrated by incubating them in a 30% sucrose solution overnight, then embedded in OCT compound. Tissue sections (10 μm thick) were obtained using a cryostat (Leica microsystems GmbH, Wetzlar, Germany), mounted onto poly-L-lysine-coated slides. The sections were air-dried for 10 min before being immersed in ice-cold acetone for 20 min. They were then air-dried again and stored at -20°C. For staining, the sections were rehydrated with PBS and blocked with 3% BSA in PBS at room temperature for 20 min. The sections were incubated in hematoxylin solution for 5 min, followed by eosin (1%) staining for 30 min. After dehydration in an ethanol solution, the samples were embedded in 60% glycerol and covered with a coverslip. The samples were analyzed using the EVOS™ M7000 imaging system (ThermoFisher Scientific, Waltham, MA, USA) at Ewha Fluorescence Core Imaging Center.

### Flow cytometry

Single-cell suspensions (2 × 10^6^/mL) from spleens, thymi, and lymph nodes were washed, blocked using the appropriate sera, and stained for specific cell surface markers using biotin- or fluorochrome-conjugated Abs in staining buffer (0.5% BSA and 0.05% sodium azide in PBS), according to standard procedures. Cells were fixed and permeabilized for intracellular staining using a Cytofix/Cytoperm™ reagent, and intracellular cytokines were stained with fluorochrome-conjugated Abs. Data were acquired at Ewha Fluorescence Core Imaging Center using an LSR Fortessa TM cell analyzer and BD FACSDiva software v8.0.1 (BD Biosciences, San Jose, CA, USA). Light scattering and fluorescent signals were analyzed as dot plots of forward and side scatter (FSC-H and SSC-H) versus the fluorescence intensity. The cell counts in the gated area are represented graphically in the figures.

### Immunoblotting

The T cell stimulation was terminated by adding an equal volume of the ice-cold medium before the cell lysates were prepared in 1% NP-40 lysis buffer containing protease and phosphatase inhibitors, as described previously (49). Lysates (50 μg) were mixed with 2X Laemmli sample buffer, boiled, and subjected to 8% or 10% SDS-PAGE. IB was performed using the indicated Abs followed by the appropriate HRP-conjugated secondary Ab. Chemiluminescent detection was conducted using the advised ECL reagents.

### Intracellular calcium measurements

Calcium flux was measured as described previously (22). Briefly, purified mouse splenic T cells were suspended in a medium and loaded with 5 μM fura-2AM at 37°C for 1 h. The cells were washed twice with Ca^2+^ loading buffer, resuspended in Ca^2+^ loading buffer (1 × 10^6^ cells/mL), and incubated at room temperature for 20 min. After measuring baseline Ca^2+^ levels for 50 sec, the cytosolic free Ca^2+^ concentration was monitored by the addition of an anti-mouse CD3ε mAb (2 μg/mL) followed by cross-linking with a goat anti-Armenian hamster IgG (10 μg/mL), using an RF-5301 PC Spectrofluorophotometer (Shimadzu, Kyoto, Japan). Ionomycin (2 μM) was added to induce the maximum Ca^2+^ increase as a control for the fura-2AM dye loading.

### T cell activation, proliferation, and actin polymerization assays

Purified T cells (2 × 10^5^) from spleens were cultured in wells coated with anti-mouse CD3ε Ab (1 μg/mL) for 18 or 48 h to measure activation markers. The cells were cultured in Gibco™ RPMI1640 medium supplemented with 10% fetal bovine serum (FBS). The activated cells were then stained with a fluorochrome-conjugated specific Ab for CD69 or CD25 on the surface, and the fluorescence intensity was analyzed. For the cell proliferation assay, purified splenic T cells were labeled using 5 μM CFSE solution, and the fluorescence-labeled cells were cultured in a plate coated with anti-mouse CD3ε Abs (1 μg/mL). After incubation for 48 or 72 h in Gibco™ RPMI1640 medium supplemented with 10% FBS, the fluorescence intensity was analyzed. For the actin polymerization assay, purified splenic T cells (1 × 10^6^) were activated for 18 h in a plate coated with anti-mouse CD3ε Abs (5 μg/mL), fixed with 4% (v/v) PFA, permeabilized with 0.1% (v/v) Triton X-100, and stained with phalloidin-FITC before the fluorescent intensity was measured. The data were acquired at Ewha Fluorescence Core Imaging Center using an LSR Fortessa TM cell analyzer and BD FACSDiva software v8.0.1 (BD Biosciences, San Jose, CA, USA).

### ELISA

To measure mouse IL-2, IFN-γ, and IL-17, the Ready-SET-Go cytokine ELISA sets were used according to the manufacturer’s instructions. For IL-2 secretion, purified splenic T cells (2 × 10^5^) were cultured in 200 μL of medium in a 96-well U-bottomed culture plate coated with anti-mouse CD3ε Abs (1 μg/mL). After incubating the cells for 24 and 48 h, the culture supernatants were collected and added to each well of the 96-well flat-bottomed ELISA plates. For IFN-γ and IL-17 production, sera were collected from the mice immunized with MOG p33–55 for 15 days and added to each well of the 96-well flat-bottomed ELISA plates prepared for the assay. At the end of the assay, the plates were analyzed at 450 nm using a microplate reader, Spectra MAX 190 (Molecular Devices, San Jose, CA, USA). For the anti-MOG p33–55-specific IgM and IgG measurements, sera were collected from the mice immunized with MOG p33–55 for 15 days. Serially diluted serum samples were subsequently added to each well of the 96-well microtiter plates coated with MOG (10 μg/mL) in bicarbonate buffer at 37°C and blocked with 1% BSA overnight. The plates containing the serum samples were incubated at 4°C overnight, and specific binding was detected using alkaline phosphatase-labeled goat anti-mouse IgM or goat anti-mouse IgG. After the assay, the plates were measured at 490 nm using a microplate reader, Spectra MAX 190.

### Adhesion assay

Purified splenic T cells were either left unstimulated or stimulated with anti-CD3ε Abs (5 μg/mL) and anti-hamster IgG (10 μg/mL) at 37°C for 30 min. A flat-bottomed 96-well plate was coated at 4°C overnight with either fibronectin (3 μg/mL), laminin (10 μg/mL), or ICAM-1-Fc (1 μg/mL), followed by blocking with 2.5% BSA at 37°C for 2 h. Cells (2.5 × 10^5^) were added in triplicate to the wells of the plate. Afterward, the plate was incubated at 37°C for 30 min, and adherent cells were detached using 50 mM EDTA and counted. The percentage of adherent cells was then calculated.

### T cell polarization assay

Coverslips were prepared by incubating them in 10% H_2_O_2_ in 0.1N HCl, followed by coating with fibronectin (20 μg/mL) in PBS containing Mg^2+^ and Ca^2+^ at room temperature for 2 h. Purified, TCR-activated splenic T cells (1~3 × 10^5^) were added to the coverslips and incubated at 37°C for 30 min. Adherent cells were then fixed with 4% (v/v) paraformaldehyde. The adhered cells were analyzed using a Nikon Eclipse TS2R microscope along with NIS-Elements BR 5.01.00 software (Tokyo, Japan) at Ewha Fluorescence Core Imaging Center. At least 50 T cells in a selected area were blindly scored as either circular (unpolarized) or as having an obvious leading edge and uropod (polarized). Three selected areas were examined in each coverslip. The results were expressed as the percentage of polarized cells relative to the total number of adhered cells.

### GTPase activation assay

The activation of Rac1 and Rap1 GTPases was assayed as previously described (22), using the GST-RalGDS and GST-PAK1 plasmids that were kindly provided by Drs. D. D. Billadeau (Mayo Clinic, Rochester, MN) and Y. S. Bae (Ewha Womans University, Seoul, Korea), respectively. Glutathione agarose beads were conjugated with PAK1 PBD (GST-PBD) or RalGDS RBD (GST-RBD). Purified splenic T cells (5 × 10^6^) were either unstimulated or stimulated with an anti-CD3ε (5 μg/mL) and anti-hamster IgG (10 μg/mL) at 37°C. Cells were lysed in 600 μL of GTPase activation buffer at each time point after stimulation. Cell lysates (500 μL) were transferred to the glutathione agarose beads, GST-PBD and GST-RBD, for the Rac1 and the Rap1 activation assays, respectively. After incubating at 4°C for 10 minutes, the beads were mixed with 2X Laemmli reducing buffer, boiled at 96°C for 5 min, and subjected to 14% PAGE. IB was performed for Rac1 or Rap1. Loading controls for the beads were observed using an anti-GST Ab.

### Statistical analysis

Comparisons were performed between the samples using an unpaired *t*-test. Comparisons of multiple conditions were performed using two-way ANOVA with *post hoc* tests. All statistical analyses were performed using Prism 5 software (GraphPad Software Inc., San Diego, CA, USA). Values of *p* < 0.05 were considered significant. Asterisks are used to indicate significant differences between the different groups (* *p* < 0.05; ** *p* < 0.01; *** *p* < 0.001).

## Data Availability

The original contributions presented in the study are included in the article/**Supplementary Material**. Further inquiries can be directed to the corresponding author.
