# Supplementary figures and images for: T cells in ARAP-deficient mice present defective T cell receptor signaling and reduced severity in an experimentally-induced autoimmune disease
Source: Front Immunol. 2025 Apr 8;16:1556616. doi: 10.3389/fimmu.2025.1556616 (PMC12011753; doi:10.3389/fimmu.2025.1556616)

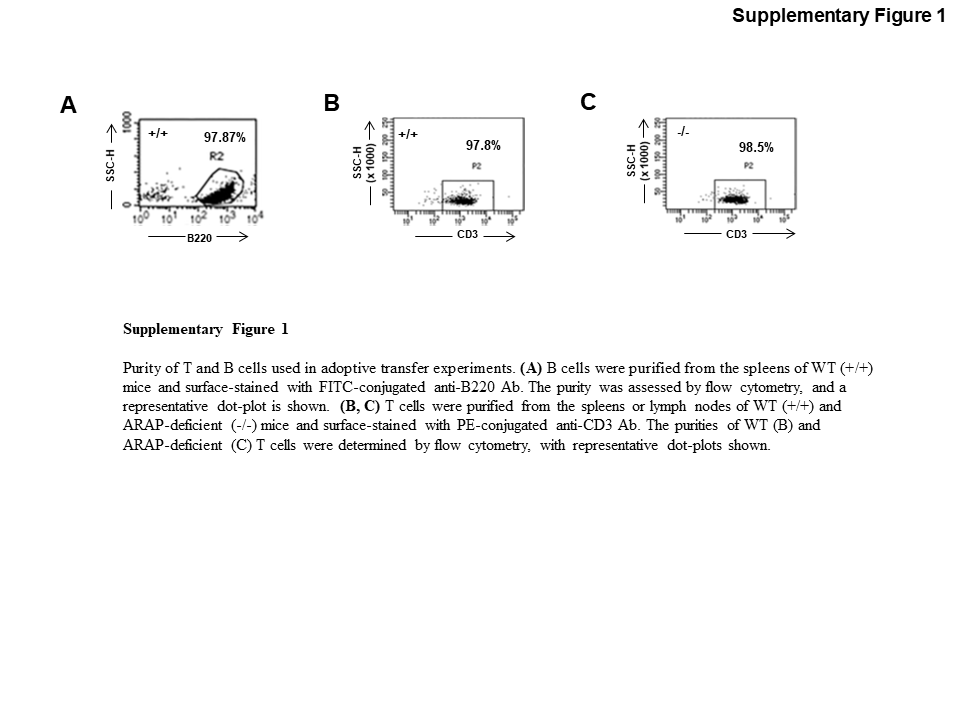

Supplement: Supplementary file 1 [file Image1.tif]
